# Peripheral temperature dysregulation associated with functionally altered Na_V_1.8 channels

**DOI:** 10.1007/s00424-023-02856-2

**Published:** 2023-09-11

**Authors:** Simon Loose, Annette Lischka, Samuel Kuehs, Carla Nau, Stefan H. Heinemann, Ingo Kurth, Enrico Leipold

**Affiliations:** 1 Department of Anesthesiology and Intensive Care & CBBM – Center of Brain, Behavior and Metabolism, University of Luebeck, Ratzeburger Allee 160, 23562 Luebeck, Germany; 2 Institute for Human Genetics and Genomic Medicine, Medical Faculty, RWTH Aachen University, Aachen, Germany; 3 Center for Molecular Biomedicine, Department of Biophysics, Friedrich Schiller University Jena and Jena University Hospital, Jena, Germany

**Keywords:** SCN10A, Sodium channel, Na_V_1.8, Channelopathy, Sensory neuropathy

## Abstract

The voltage-gated sodium channel Na_V_1.8 is prominently expressed in the soma and axons of small-caliber sensory neurons, and pathogenic variants of the corresponding gene *SCN10A* are associated with peripheral pain and autonomic dysfunction. While most disease-associated *SCN10A* variants confer gain-of-function properties to Na_V_1.8, resulting in hyperexcitability of sensory neurons, a few affect afferent excitability through a loss-of-function mechanism. Using whole-exome sequencing, we here identify a rare heterozygous *SCN10A* missense variant resulting in alteration p.V1287I in Na_V_1.8 in a patient with a 15-year history of progressively worsening temperature dysregulation in the distal extremities, particularly in the feet. Further symptoms include increasingly intensifying tingling and numbness in the fingers and increased sweating. To assess the impact of p.V1287I on channel function, we performed voltage-clamp recordings demonstrating that the alteration confers loss- and gain-of-function characteristics to Na_V_1.8 characterized by a right-shifted voltage dependence of channel activation and inactivation. Current-clamp recordings from transfected mouse dorsal root ganglion neurons further revealed that Na_V_1.8-V1287I channels broaden the action potentials of sensory neurons and increase their firing rates in response to depolarizing current stimulations, indicating a gain-of-function mechanism of the variant at the cellular level in a heterozygous setting. The data support the hypothesis that the properties of Na_V_1.8 p.V1287I are causative for the patient’s symptoms and that nonpainful peripheral paresthesias should be considered part of the clinical spectrum of Na_V_1.8-associated disorders.

## Introduction

The voltage-gated sodium (Na_V_) channel Na_V_1.8, encoded by *SCN10A*, is preferentially expressed in dorsal root ganglion (DRG) and trigeminal ganglion neurons and is present in cutaneous nerve terminals and in the cornea [1, 3, 48]. Among the nine mammalian Na_V_ channels (Na_V_1.1–1.9), Na_V_1.8 is unique with respect to its gating properties. Compared to other Na_V_ subtypes, it activates and inactivates at more depolarized voltages, exhibits a slow rate of inactivation, and rapidly recovers from inactivation [1, 52]. Because of these properties, Na_V_1.8 mediates most of the Na^+^ inward current during the upstroke of the action potential in sensory afferents and supports high-frequency firing of these neurons as required for the transmission of sensory information [4, 27, 51]. In addition, owing to its relative resistance to cold-induced channel inactivation, Na_V_1.8 is important for action potential propagation at low temperatures [62].

Several pathogenic or likely pathogenic missense variants in *SCN10A* have been linked to peripheral neuropathies characterized by recurrent pain episodes and itching in distal body parts, predominantly involving the feet and lower legs [19, 28, 35]. Affected individuals often present with reduced intraepidermal nerve fiber density and elevated thermal thresholds being compatible with the clinical diagnosis of small fiber neuropathy (SFN). Consistent with the pain phenotype of the patients, associated variants have been demonstrated to render sensory neurons hyperexcitable by conferring gain-of-function attributes to Na_V_1.8, such as enhanced channel activation [19, 35] or impaired channel inactivation [28]. However, variants causing mild loss-of-function of Na_V_1.8 have also been linked to human pain phenotypes. For example, Kist et al. identified variant p.M650K in a patient diagnosed with erythromelalgia, a condition characterized by spontaneous peripheral pain and characteristic reddening and warming of affected body parts. As revealed by patch-clamp recordings, this alteration reduces the activity of Na_V_1.8 by enhancing channel inactivation [40]. Another *SCN10A* loss-of-function variant (p.D1639N) was identified in a patient suffering from diffuse pain and gastroparesis [9] and was shown to impair the trafficking of Na_V_1.8 to the cell surface [38]. A more frequent variant harboring the non-synonymous amino acid substitution p.A1073V in Na_V_1.8 has been suggested to reduce mechanical pain sensitivity in humans [15]. Relatedly, homozygosity of this variant was more frequently identified in patients with hypoalgesic inflammatory bowel disease [25] and in patients with reduced severity of postoperative abdominal pain after sigmoid colectomy [6].

In this study, we examine the Na_V_1.8 variant p.V1287I identified in a patient with discomfort due to progressive temperature regulation disturbances affecting hands and feet. The variant channels exhibit a mixture of loss- and gain-of-function properties caused by right-shifted voltage dependence of channel activation and inactivation, respectively. Furthermore, Na_V_1.8-V1287I channels render sensory neurons hyperexcitable and affect the shape of action potentials.

## Materials and methods

### Patient consent

The study was approved by the local research ethics committee or institutional review board of the participating institutions (EK302-16). Informed consent was obtained from the participating patient before study initiation.

### Clinical assessment

The patient’s clinical data was assessed using a standardized in-house survey for the investigation of pain disorders. Routine nerve conduction studies were performed to exclude a large fiber polyneuropathy as underlying cause.

### Whole-exome sequencing

Whole-exome sequencing (WES) was performed on DNA from peripheral blood using Illumina Nextera Rapid Capture Exome, version 1.2, for enrichment. The respective libraries were sequenced on an Illumina NextSeq 500 sequencer. Alignment and variant calling were performed using SeqMule (version 1.2). KGGSeq (version 1.0, April 14, 2017) was used for the annotation of the resulting variant files and variants with a minor allele frequency >0.75% in public databases were excluded, yielding variant NM_006514.4:c.3859G>A in *SCN10A*. The identified variant was confirmed by Sanger sequencing. No other obviously pathogenic and phenotypically matching variant was found.

### Generation of channel mutants

A pCMV6-XL5 expression plasmid containing the human Na_V_1.8 cDNA (*SCN10A*, NM_006514.2) was purchased from OriGene Technologies Inc. (Rockville, MD, USA). Alterations p.V1287I and p.V1287A were introduced in the coding sequence of Na_V_1.8 using a PCR-based strategy. The coding sequence of all expression constructs was verified by Sanger sequencing.

### Isolation and transfection of mouse dorsal root ganglion neurons

Animal care and associated experimental procedures followed the guidelines established by the animal welfare committee of the University of Luebeck.

For voltage-clamp recordings, dorsal root ganglia from all levels of the spinal cord of 8–12 weeks old homozygous *Scn10a*^−/−^/*Scn11a*^−/−^ double-knockout mice (strain C57BL6) lacking functional Na_V_1.8 and Na_V_1.9 channels (Na_V_1.8/Na_V_1.9 DKO mice) were extracted following a procedure described earlier [11]. Na_V_1.8/Na_V_1.9 DKO mice were generated by disrupting the genes *Scn10a* and *Scn11a* in *cis* using a CRISPR-Cas approach. The generation of the Na_V_1.8/Na_V_1.9 DKO mouse line will be published elsewhere. Isolated Na_V_1.8/Na_V_1.9 DKO DRG neurons were transfected by electroporation using a 4D-Nucleofector™ (Lonza, Basel, Switzerland) with the P3 Primary Cell 4D-Nucleofector™ X Kit S (V4XP-3032) according to a previously described procedure [42]. Briefly, purified DRG neurons from each animal were resuspended in 20 μl of P3 Primary Cell Solution containing Supplement 1, 1.5 μg of a plasmid encoding either human Na_V_1.8, Na_V_1.8-V1287I, or Na_V_1.8-V1287A, and 0.3 μg of a plasmid encoding the enhanced green fluorescent protein (EGFP). Transfection was performed using the electroporation protocol CA137 of the 4D-Neucleofector™. After electroporation, 150 μl of low-calcium RPMI (Roswell Park Memorial Institute; Invitrogen) 1640 medium was added to each cell suspension, and cells were allowed to recover for 10 min in a 5% CO_2_ incubator at 37 °C. The cell suspensions were then diluted in 300 μl DRG medium and immediately seeded on poly-D-lysin-coated glass coverslips, which were placed in 24-well plates containing 1 ml DRG medium per well. DRG medium contained 89.5% DMEM/F12 (Dulbecco’s Modified Eagle’s Medium with Ham’s F12; Invitrogen) supplemented with 9.5% fetal bovine serum and 1% penicillin/streptomycin (Invitrogen).

For action-potential recordings, DRGs were extracted from all levels of the spinal cord of 8–12 weeks old wild-type C57BL6 mice and processed as described above, except that purified DRG neurons from each animal were divided into two equal lots to enable experiments with wild-type and mutant Na_V_1.8 channels using the same batch of cells [42]. Both cell aliquots were resuspended individually in 20 μl P3 Primary Cell Solution containing Supplement 1 and 0.3 μg of a plasmid encoding EGFP. Subsequently, one sample was supplemented with 1.5 μg of a Na_V_1.8-encoding plasmid, while the second sample was supplemented with 1.5 μg of a plasmid encoding either mutant Na_V_1.8-V1287I or Na_V_1.8-V1287A. Electroporation, cell seeding, and cultivation were as described above.

Transfected cells were incubated in a 5% CO_2_ incubator at 37 °C and used for electrophysiological experiments 24–48 h after transfection. Voltage-clamp and current-clamp recordings were restricted to successfully transfected small-diameter (<25 μm) DRG neurons, identified by their green florescence using an AF-1850 LED light source and a GFP filter set.

### Electrophysiology

Voltage and current recordings were obtained in the whole-cell configuration of the patch-clamp method using an EPC-10 patch-clamp amplifier operated by PatchMaster software (HEKA Elektronik, Lambrecht, Germany). Patch pipettes were fabricated from Kimax borosilicate glass of about 1.0–2.5 MΩ resistance and coated with RTV silicone adhesive (Dow Corning GmbH, Wiesbaden, Germany) to reduce tip capacitance. Series resistance was corrected electronically up to 90% and all voltages were corrected for the liquid junction potential (−7 mV). Bath solution for voltage-clamp recordings contained (in mM) 130 NaCl, 2 KCl, 2.5 CaCl_2_, 1 MgCl_2_, 20 tetraethylammonium chloride (TEA-Cl), and 10 HEPES (pH 7.4 with NaOH) and was supplemented with tetrodotoxin (1 μM), CdCl_2_ (100 μM), and 4-aminopyridine (1 mM) to block endogenous Na^+^ currents, Ca^2+^ currents, and K^+^ currents, respectively. The pipette contained (in mM) 10 NaCl, 130 CsF, 10 EGTA, and 10 HEPES (pH 7.3 with CsOH). The bath solution for current-clamp recordings contained (in mM) 120 NaCl, 3 KCl, 2.5 CaCl_2_, 1 MgCl_2_, 30 HEPES, and 15 glucose (pH 7.4 with NaOH), and the pipette 125 KCl, 8 NaCl, 1 CaCl_2_, 1 MgCl_2_, 0.4 Na_2_-GTP, 4 Mg-ATP, 10 EGTA, and 10 HEPES (pH 7.3 with KOH). Recordings were performed at a constant temperature of 20 ± 0.5 °C using a microincubation stage (ALA Scientific Instruments, Farmingdale, NY, USA) feedback-controlled by a PTC-10 temperature controller (NPI Electronic GmbH, Tamm, Germany).

For voltage-clamp experiments with Na_V_1.8/Na_V_1.9 DKO neurons, the holding potential was set to −117 mV. Data were low-pass filtered at 5 kHz and sampled at an interval of 40 μs. Leak and capacitive currents were subtracted using a P/4 protocol with a leak holding potential of −117 mV.

#### Channel activation

Channel activation was measured with 80-ms test pulses ranging from −107 to 53 mV applied in steps of 10 mV every 3 s. The voltage dependence of channel activation was estimated from peak current densities using a Hodgkin-Huxley activation model involving *m* = 3 activation gates and a linear single-channel conductance:

1 $$\frac{I(V)}{C_m}=\Gamma \cdot \left(V-{E}_{rev}\right)\cdot \frac{1}{{\left(1+{e}^{-\left(V-{V}_m\right)/{k}_m}\right)}^3}$$

with the cell membrane capacitance *C*_*m*_, conductance density *Γ*, and the reversal potential *E*_*rev*_. *V*_*m*_ is the half-maximal activation voltage per gate and *k*_*m*_ the corresponding slope factor.

#### Gating kinetics

The kinetics of activation and fast inactivation of Na_V_1.8 wild-type and mutant channels were estimated by fitting current responses to depolarizing voltage steps according to the following formalism:

2 $$I(t)={I}_o\cdot {\left(1-{e}^{-\frac{t}{\tau_a}}\right)}^3\cdot \left({h}_{\infty }+\left(1-{h}_{\infty}\right)\cdot {e}^{-\frac{t}{\tau_h}}\right)$$

with the current amplitude *I*_0_, the time constant for activation *τ*_*a*_, the fraction of non-inactivating channels *h*_*∞*_, and the time constant *τ*_*h*_ characterizing fast channel inactivation. Kinetics of channel deactivation was estimated with single-exponential fits yielding the deactivation time constant *τ*_*d*_. The voltage dependence of activation and deactivation time constants was described with a one-step activation/deactivation model:

3 $$\tau (V)={\tau}_0+\frac{1}{a_0+\left({e}^{\frac{\left(V-{V}_m\right)\cdot q\cdot \delta }{kT}}+{e}^{\frac{\left(V-{V}_m\right)\cdot q\cdot \left(1-\delta \right)}{kT}}\right)}$$

*τ*_0_ is the voltage-independent, limiting speed of deactivation, and *α*_0_ is the rate at the equilibrium voltage *V*_*m*_, and *kT* is the thermal energy. *q* is the total gating charge transfer and *δ* is the symmetry factor specifying the gating charge fraction associated to channel activation.

#### Steady-state inactivation

Wild-type and mutant Na_V_1.8 channels were activated with a first 40-ms test pulse to −7 mV followed by a conditioning interval of 500 ms at voltages ranging from −117 to 13 mV in steps of 10 mV. Peak currents of not inactivated channels were measured in a subsequent 40-ms test pulse to −7 mV. The repetition interval was 5 s. The current amplitude after conditioning (*I*_500_) normalized to the control current amplitude before conditioning (*I*_0_) was described with a Boltzmann formalism:

4 $$\frac{I_{500}(V)}{I_0}={h}_{max}-\left({h}_{max}-{h}_{min}\right)\cdot \left(\frac{1}{1+{e}^{-\left(V-{V}_h\right)/{k}_h}}\right)$$

with the maximal and minimal channel availability *h*_*max*_ and *h*_*min*_, the half-maximal inactivation voltage *V*_*h*_, and the corresponding slope factor *k*_*h*_.

#### Current-clamp recordings

The resting membrane voltage (RMP) of transfected DRG neurons was measured by zero current injection directly after establishing the whole-cell configuration. Single action potentials were evoked by injecting a current of 100–200 pA for a period of 10 ms, followed by a 200-ms period without current injection. Sampling interval for voltage measurements was 50 μs. Parameters characterizing individual action potentials, including action potential peak voltage (*V*_*peak*_), minimum after-hyperpolarization voltage (*V*_*AHP*_), voltage threshold of action potential firing (*V*_*th*_), and action potential duration measured at a voltage of 0 mV (*Width*_0_), were analyzed with IgorPro software using customized scripts. *V*_*th*_ was defined as voltage at which *dV*/*dt* reached the level of 0.03 * (*dV*/*dt*_*max*_ − *dV*/*dt*_*min*_) + *dV*/*dt*_*min*_ [43]. Trains of action potentials were evoked repetitively by 2-s current injections, ranging from 0 to 140 pA in steps of 20 pA. Mean firing frequencies were determined using 0 mV as action potential detection threshold.

Data were analyzed with FitMaster (HEKA Elektronik) and IgorPro 8 (WaveMetrics, Lake Oswego, OR, USA) software. Data are presented as means ± s.e.m., (*n*) with *n* being the number of independent experiments. Statistical comparisons of two groups of data were made using the two-tailed Student’s *t*-test; *P* values are given explicitly when appropriate.

## Results

### Clinical assessment and genetic testing

A 43-year-old male patient presented with a slowly progressive peripheral temperature regulation disorder that started insidiously at the age of 30. The symptoms were limited to the hands and feet; other parts of the body were not affected. The patient reported a strong overheating of his feet in warm weather, while in cold weather they would hardly reach a minimum pleasant temperature. Especially periods of overheating were described by the patient as difficult to bear, resulting in increasing discomfort, even despite the absence of pure pain. The symptoms were accompanied by severe reddening of the skin and could last for hours. Over time, the patient additionally noticed increasing sweating as well as tingling and numbness in his fifth fingers.

The quality of life was strongly compromised during the first years after disease onset. However, the patient was able to develop strategies to avoid triggering temperature and thus avoid the unpleasant situations. These strategies include, for example, wearing gloves when cycling even when the outside temperature is 15 °C and wearing several pairs of socks or exercising when feet start to cool down. Because strategies to reverse overheating, such as active cooling of affected extremities, are far less effective, he consistently avoids heat. No treatment with medication had been started. Nerve conduction studies indicated no abnormalities, excluding a large fiber neuropathy. The family history did not reveal evidence of other similarly affected family members; however, relatives of the patient were not available for clinical examination or genetic testing.

Whole-exome sequencing (WES) revealed heterozygosity for the rare missense variant NM_006514.4:c.3859G>A in exon 23 of the *SCN10A* gene encoding the voltage-gated sodium channel Na_V_1.8. No additional pathogenic or likely pathogenic variants in other genes associated with peripheral sensory disorders, for example, *SCN9A*, *SCN11A*, or *TRPV1*, were detected. The *SCN10A* variant identified in the patient is reported in 167 of 282,600 alleles in the current version of the population database gnomAD (gnomAD v2.1.1). It is enriched in the European (non-Finnish) population (149x) but is very rare in other populations. In addition, the clinical database ClinVar contains conflicting reports regarding the pathogenicity of the variant, including classifications such as benign, likely benign, and variant of uncertain clinical significance (VUS) (https://www.ncbi.nlm.nih.gov/clinvar/variation/95402/?new_evidence=true).

**Fig. 1:**
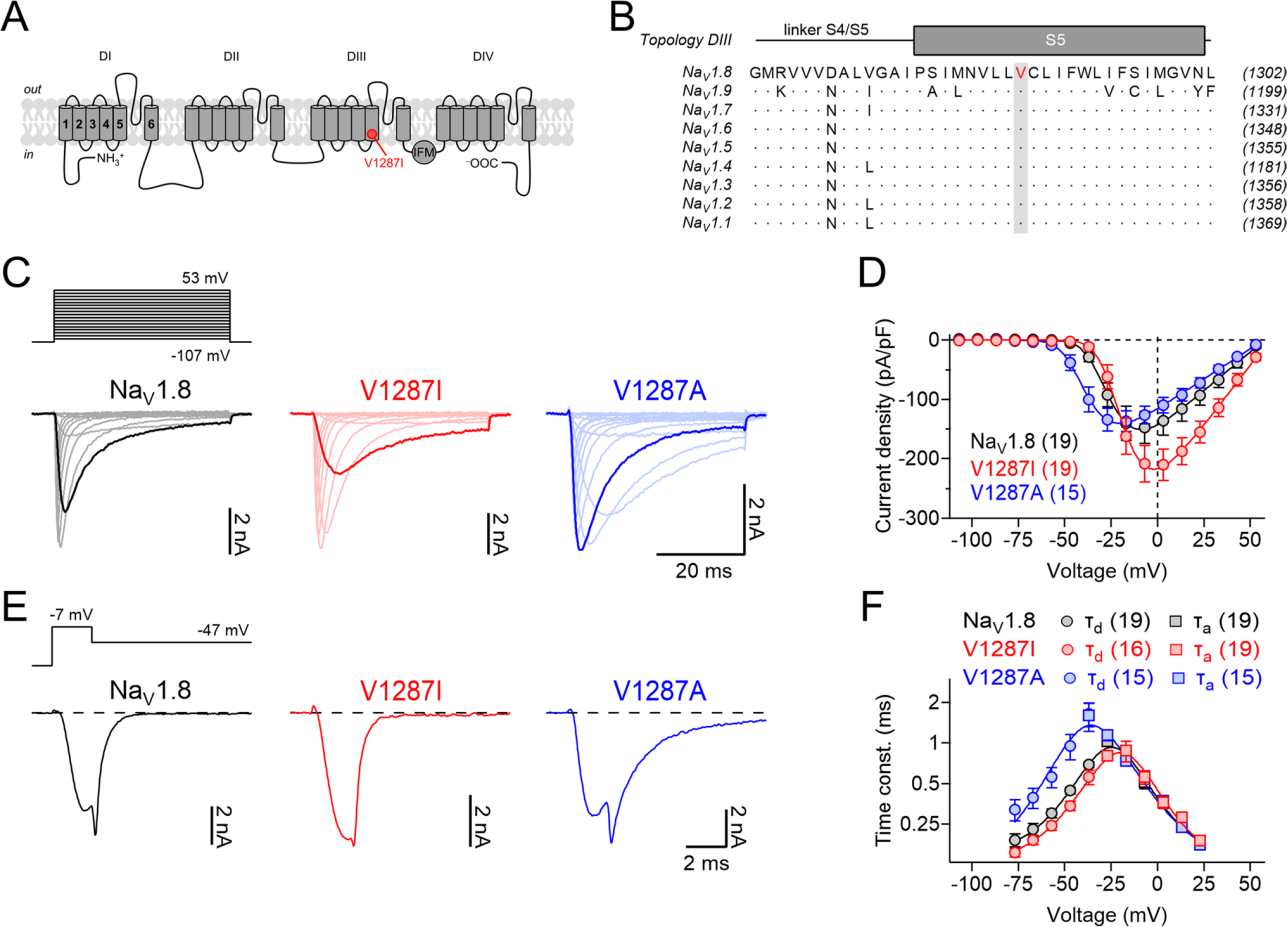
Alteration p.V1287I accelerates deactivation of Na_V_1.8 channels. **A** Membrane topology of the alpha subunit of Na_V_1.8 with the four homologous domains DI–DIV. The position of alteration p.V1287I in transmembrane segment S5 in DIII is highlighted in red. **B** Amino acid sequence alignment of the human Na_V_1 isoforms (Na_V_1.1–1.9) covering transmembrane segment S5 in DIII of the channels. The affected valine at position 1287 is shown red in the Na_V_1.8 sequence. Dots indicate sequence conservation with respect to Na_V_1.8. Numbers in parentheses refer to the positions of the last residue shown with respect to the full-length protein sequences. **C** Representative whole-cell current traces recorded from Na_V_1.8/Na_V_1.9 DKO DRG neurons, expressing human Na_V_1.8 (black), Na_V_1.8-V1287I (red), or Na_V_1.8-V1287A (blue), in response to depolarizations ranging from −107 mV to 53 mV in steps of 10 mV. Current responses obtained at a voltage of −27 mV are shown as thick lines. The corresponding pulse protocol is shown on top. **D** Peak current densities from experiments as shown in **C**, plotted as a function of voltage. Superimposed fits describe the voltage dependence of channel activation according to Eq. (1). **E** Representative whole-cell tail currents of Na_V_1.8 wild-type and mutant channels in response to a hyperpolarizing voltage step from −7 to −47 mV (*top*) to characterize channel deactivation. **F** Time constants describing gate activation (*τ*_*a*_, squares) and channel deactivation (*τ*_*d*_, circles) obtained from experiments as shown in **C** and **E**, plotted as a function of voltage. Superimposed curves are data fits according to Eq. (3) characterizing the voltage dependence of activation and deactivation kinetics of wild-type Na_V_1.8 (black) as well as variants p.V1287I (red) and p.V1287A (blue). Data points in **D** and **F** represent means ± s.e.m. with numbers of experimental replicates provided in parentheses

The variant affects a highly conserved valine residue (p.V1287I) located in the intracellular portion of transmembrane segment S5 in domain III of Na_V_1.8 (Fig. 1A, B), a region previously shown to be involved in inactivation of Na_V_ channels [49, 55]. More importantly, rare variants that replace the conserved valine with alanine in the homologous positions of Na_V_1.7 (p.V1613A) [59] and Na_V_1.9 channels (p.V1184A) [42], both expressed in sensory neurons, have been causally linked with primary erythromelalgia and cold-aggravated peripheral pain episodes, respectively, suggesting that alteration p.V1287I likely alters functional properties of Na_V_1.8 and may be related to the patient’s symptoms.

### Evaluation of Na_V_1.8 mutant channels in voltage-clamp experiments

For assessing the impact of variant p.V1287I on channel function, whole-cell voltage-clamp experiments were performed in murine Na_V_1.8/Na_V_1.9 DKO DRG neurons transfected with expression plasmids encoding either human wild-type Na_V_1.8 or Na_V_1.8-V1287I channels. Because variants that replace the conserved valine in Na_V_1.7 (p.V1316A) and Na_V_1.9 (p.V1184A) with alanine have previously been associated with peripheral pain disorders [42, 59], we also generated and functionally characterized Na_V_1.8 variant p.V1287A. All recordings were obtained in the presence of tetrodotoxin (TTX, 1 μM) to quantitatively inhibit TTX-resistant Na^+^ currents endogenous to Na_V_1.8/Na_V_1.9 DKO DRG neurons, thereby facilitating the functional analysis of human Na_V_1.8 in these cells.

Both wild-type and p.V1287I channels produced robust Na^+^ currents in Na_V_1.8/Na_V_1.9 DKO DRG neurons with activation and inactivation kinetics typical for Na_V_1.8 (Fig. 1C). Compared to wild-type, variant p.V1287I yielded larger inward current densities particularly at high membrane voltages, being significant between −3 and 53 mV (*P* < 0.05). Analysis of the voltage dependence of peak current densities (Fig. 1D) and the voltage dependence of activation and deactivation kinetics (Fig. 1E, F) revealed that alteration p.V1287I accelerated channel deactivation compatible with a right-shift of the half-maximal gate activation voltage, *V*_*m*_, by 8.6 mV (Na_V_1.8: −35.6 ± 1.2 mV, p.V1287I: −27.0 ± 1.1 mV, *P* < 0.001). The associated slope factor, *k*_*m*_, reflecting the voltage dependence of channel activation, was not affected by p.V1287I (Na_V_1.8: −10.1 ± 0.6 mV, p.V1287I: −9.5 ± 1.0 mV, *P* > 0.05). The voltage dependence of activation and deactivation kinetics (Fig. 1F) of wild-type and p.V1287I channels were characterized by a gating charge transfer of 3.91 ± 0.52 e_0_ and 3.60 ± 0.49 e_0_ and corresponding symmetry factors specifying the charge fraction associated to channel activation, of 0.48 ± 0.06 and 0.47 ± 0.06, respectively. Thus, variant p.V1287I destabilizes the open-state of Na_V_1.8 by augmenting the channel’s closing rate.

In contrast to variant p.V1287I in Na_V_1.8, disease-associated valine to alanine substitutions at the homologous positions in Na_V_1.7 (p.V1316A) and Na_V_1.9 (p.V1184A) facilitate opening of these channels, as revealed by substantial left-shifts of the voltage dependence of channel activation (approximately −6.8 mV for Na_V_1.7-V1316A and −19.5 mV for Na_V_1.9-V1184A) [42, 59]. Therefore, we also analyzed the valine to alanine substitution in the Na_V_1.8 background.

As shown in Fig. 1C–D, mutant Na_V_1.8-V1287A yielded larger inward current densities than wild-type or variant p.V1287I specifically at low voltages, being significant between −57 and −37 mV (*P* < 0.05). Unlike alteration p.V1287I which accelerated deactivation of Na_V_1.8 and right-shifted *V*_*m*_, mutation p.V1287A slowed down channel deactivation (Fig. 1E–F) and left-shifted *V*_*m*_ by −12.1 mV (−47.7 ± 2.0 mV, *P* < 0.001) (Fig. 1D). The corresponding slope factor *k*_*m*_ was not affected by this mutation (8.8 ± 0.9 mV, *P* > 0.05). The voltage dependence of activation and deactivation kinetics of p.V1287A channels was compatible with a charge transfer of 3.44 ± 0.45 e_0_ and an associated symmetry factor of 0.48 ± 0.17.

**Fig. 2:**
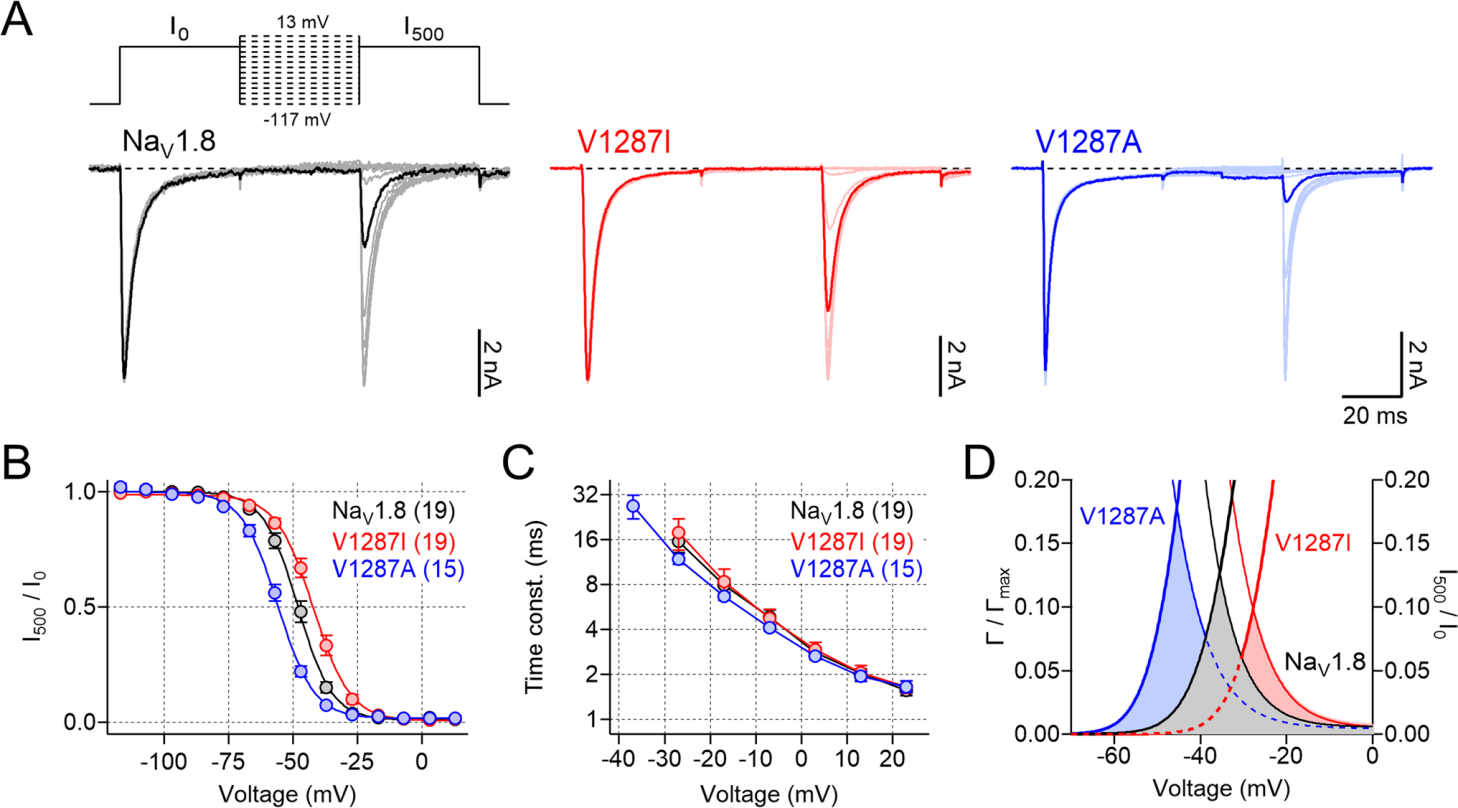
Analysis of fast channel inactivation. **A** Families of whole-cell current traces recorded from Na_V_1.8/Na_V_1.9 DKO neurons expressing Na_V_1.8 (black), Na_V_1.8-V1287I (red), or Na_V_1.8-V1287A (blue) before (*I*_0_) and after (*I*_500_) a 500-ms conditioning period ranging from −127 to 13 mV in steps of 10 mV. The corresponding pulse protocol is shown on top. Current responses belonging to a conditioning voltage of −47 mV are shown as thick lines. **B** Voltage dependence of steady-state fast inactivation of wild-type and mutant Na_V_1.8 channels, analyzed according to Eq. (4). **C** Single-exponential time constants characterizing inactivation kinetics of Na_V_1.8 (black), Na_V_1.8-V1287I (red), and Na_V_1.8-V1287A (blue), plotted as a function of voltage (bottom). Straight lines connect data points. **D** Boltzmann functions representing the voltage dependences of activation (*Γ*/*Γ*_*max*_, thick lines) and inactivation (*I*_500_/*I*_0_, thin lines) of wild-type Na_V_1.8 (black) as well as mutants p.V1287I (red) and p.V1287A (blue). Colored areas indicate overlap of activation and inactivation curves. Data points in **B** and **C** are means ± s.e.m. with *n* indicated in parentheses

The voltage dependence of steady-state channel inactivation, measured after 500-ms conditioning periods at various voltages, was also differentially affected by the two mutations (Fig. 2A, B). The disease-associated change p.V1287I right-shifted the half-maximal voltage of channel inactivation *V*_*h*_ by 5.2 mV, effectively increasing functional availability of the channels whereas mutation p.V1287A left-shifted *V*_*h*_ by −8.7 mV, thus decreasing channel availability (Na_V_1.8: −47.1 ± 0.9 mV; p.V1287I: −41.9 ± 1.3 mV, *P* < 0.01; p.V1287A: −55.8 ± 1.3 mV, *P* < 0.001). The corresponding slope factors, *k*_*h*_, characterizing the voltage dependence of steady-state inactivation of wild-type (Na_V_1.8: 5.9 ± 0.2 mV) and mutant channels (p.V1287I: 6.3 ± 0.3 mV; p.V1287A: 6.7 ± 0.3 mV; both *P* > 0.05), were not significantly different. The kinetics of fast channel inactivation was analyzed by fitting current responses as shown in Fig. 1C with single-exponential functions according to Eq. 2. In the entire voltage range explored (from −37 mV to 23 mV), neither of the two amino acid substitutions affected the inactivation kinetics of Na_V_1.8 (Fig. 2C).

The data shown so far thus demonstrate that disease-associated p.V1287I channels activate and inactivate at more positive membrane potentials, whereas activation and inactivation gating of p.V1287A channels is shifted toward more negative potentials (Fig. 2D).

### Impact of Na_V_1.8 variant p.V1287I on DRG neuron excitability

**Fig. 3:**
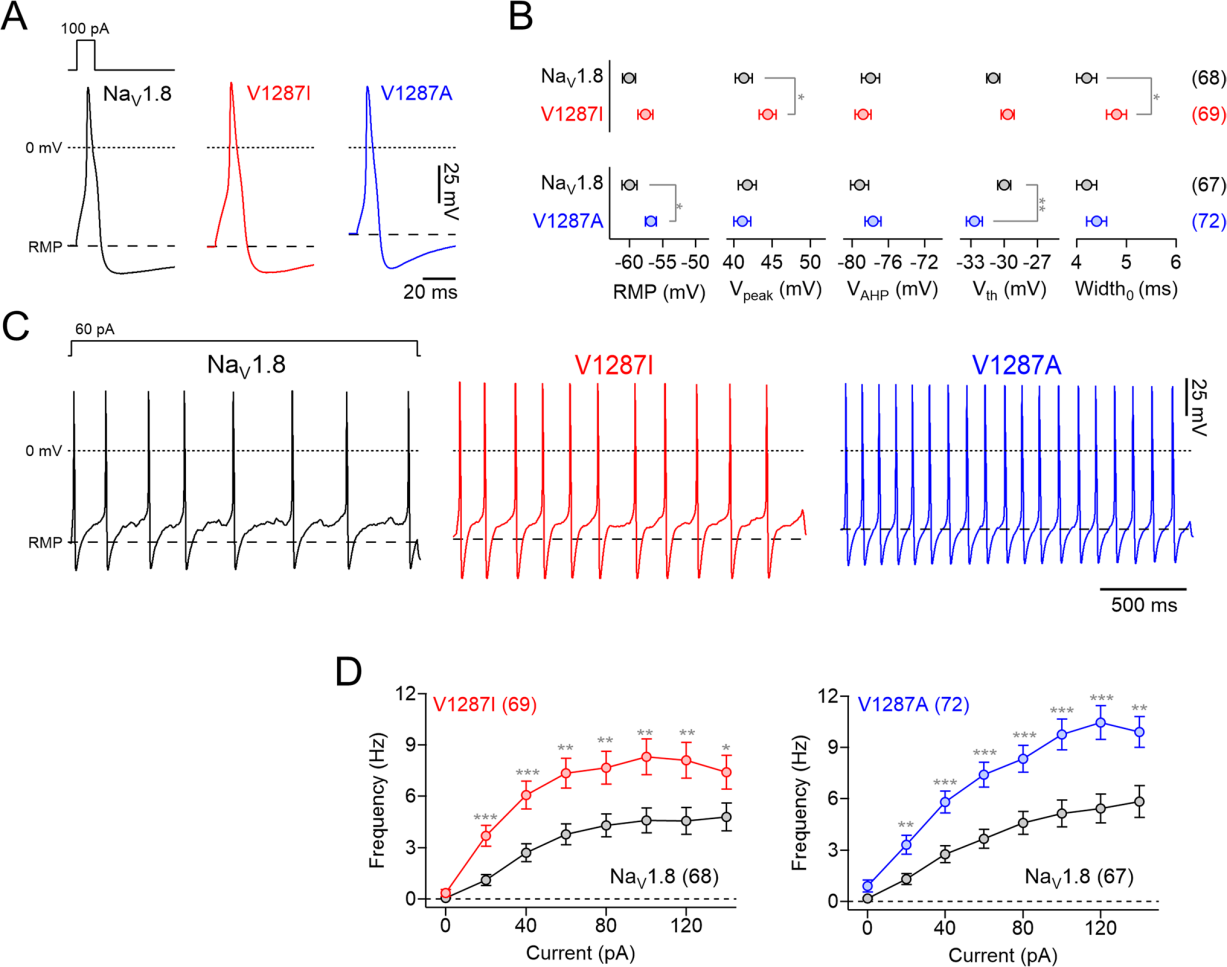
Na_V_1.8 variants render DRG neurons hyperexcitable. **A** Representative evoked action potentials in murine DRG neurons expressing human Na_V_1.8 (black), Na_V_1.8-V1287I (red), or Na_V_1.8-V1287A (blue), in response to 10-ms current injections of 100 pA (top). Dotted lines mark 0 mV; dashed lines indicate the level of the resting membrane potential (RMP). **B** Parameters characterizing action potential properties. RMP resting membrane potential, *V*_*peak*_ action potential peak voltage, *V*_*AHP*_ maximal after-hyperpolarization voltage, *V*_*th*_ action potential threshold voltage, *Width*_0_ action potential duration at 0 mV. **C** Representative trains of action potentials recorded in murine DRG neurons transfected with wild-type Na_V_1.8 (black), mutant p.V1287I (red), or mutant p.V1287A, in response to 2-s current injections of 60 pA. Dotted lines mark 0 mV; dashed lines indicate the level of the resting membrane potential (RMP). **D** Action potential frequencies as a function of injected current obtained from experiments as shown in **C**. Data in **B** and **D** are means ± s.e.m. with the numbers of neurons analyzed, *n*, indicated in parentheses. Datasets are based on cells obtained from a total of 8 (Na_V_1.8 vs p.V1287I) or 9 (Na_V_1.8 vs p.V1287A) animals. Significance between pairs of data was tested using a two-sided Student’s *t*-test: *P < 0.05; **P < 0.01

To assess the effects of p.V1287I and p.V1287A on neuronal excitability, we performed current-clamp recordings on small-diameter (<25 μm) DRG neurons isolated from wild-type mice and transfected with cDNA encoding either human Na_V_1.8 wild-type or one of the two mutant channels. To facilitate the analysis of individual action potential waveforms, single spikes were evoked with 10-ms current injections (Fig. 3A). Overexpression of variant p.V1287I increased the peak voltage of the action potential, *V*_*peak*_, by 3.1 mV (Na_V_1.8: 41.3 ± 1.1 mV; p.V1287I: 44.4 ± 1.1 mV; *P* < 0.05) and prolonged the duration of action potentials *Width*_0_ (Na_V_1.8: 4.2 ± 0.2 ms; p.V1287I: 4.8 ± 0.2 ms; *P* < 0.05). The resting membrane potential (RMP), the maximal after-hyperpolarization voltage (*V*_*AHP*_), and the threshold voltage for action potential firing (*V*_*th*_) were not significantly different between neurons expressing Na_V_1.8 wild-type or Na_V_1.8-V1287I. In contrast, overexpression of Na_V_1.8-V1287A channels significantly increased the RMP of neurons by 3.2 mV (Na_V_1.8: −60.0 ± 1.1 mV; p.V1287A: −56.8 ± 0.8 mV; *P* < 0.05) and lowered the firing threshold *V*_*th*_ from −30.0 ± 0.6 to −32.3 ± 0.7 mV (*P* < 0.01); all other parameters were unaffected (Fig. 3B).

To determine the impact of mutant channels on repetitive action potential firing, we analyzed the firing rates of transfected neurons in response to 2-s current injections increasing from 0 to 140 pA in steps of 20 pA. Representative trains of action potentials evoked by 60-pA current injections in neurons expressing Na_V_1.8 wild-type, p.V1287I, or p.V1287A channels are shown in Fig. 3C. Systematic assessment of firing frequencies as a function of injected current (Fig. 3D) revealed that neurons transfected with p.V1287I or p.V1287A channels consistently fired more action potentials compared with neurons transfected with Na_V_1.8 wild type, demonstrating that both channel variants render DRG neurons hyperexcitable.

## Discussion

Small fiber neuropathy (SFN) is a disorder affecting small-diameter nerve fibers including sensory Aδ fibers and C fibers, which mediate thermal and pain sensation, as well as autonomic functions [44]. Consequently, SFN-associated symptoms can be numerous, including peripheral pain episodes of variable severity, impaired temperature perception, itch, numbness, and deregulated sweating [50].

In this study, we identified the heterozygous missense variant p.V1287I in sensory neuron-specific Na_V_1.8 channels in a 43-year-old patient with progressive sensory discomfort manifesting as intense spontaneous flushing and, less frequently, hypothermia of the body extremities, predominantly in the feet. In particular, overheating of the feet, which can already be triggered by normal walking is difficult to reverse even by active cooling of the affected areas. Based on clinical assessment, including inconspicuous nerve conduction studies, a disorder of small nerve fibers was assumed. Although pain is considered a frequent symptom in SFN, the patient described the attacks as very unpleasant peripheral temperature dysregulation. The symptoms were rated significantly limiting quality of life without being explicitly painful. Further diagnostic tests that could confirm the involvement of small fibers, such as quantitative sensory tests or skin biopsies, have been rejected.

To date, several pathogenic variants altering the functional properties of Na_V_1.8 have been identified and causally linked to SFN, with symptoms including peripheral pain episodes, altered thresholds for warm and cold sensation, and discomfort such as itching [19, 28, 35]. The majority of these alterations confer gain-of-function properties to Na_V_1.8 by either shifting the voltage dependence of channel activation to more negative voltages [19, 35], thereby facilitating channel opening, or shifting the voltage dependence of inactivation to more positive potentials, effectively increasing the fraction of channels available for opening [28]. Consistent with these pro-excitatory properties, all these mutations increase the firing rates of sensory neurons in response to graded stimulation intensities. Although less intuitive, partial loss-of-function of Na_V_1.8 due to enhanced inactivation [40] or impaired membrane trafficking of the channel protein [38] has also been reported to underlie painful SFN in human. In addition, painful peripheral neuropathies as well as congenital pain insensitivity have been associated with mutations affecting channel subtypes Na_V_1.7 and Na_V_1.9, both of which are highly expressed in sensory neurons along with Na_V_1.8 [26]. For example, biallelic loss-of-function mutations in *SCN9A*, the gene encoding Na_V_1.7, cause congenital insensitivity to a wide range of painful stimuli [7, 24] by impairing synaptic transmission from primary nociceptor terminals to secondary neurons in the spinal cord [45]. By contrast, gain-of-function mutations of Na_V_1.7 result in hyperexcitability of sensory neurons and underlie severe neuropathic pain conditions such as primary erythromelalgia (PE) and paroxysmal extreme pain disorder (PEPD) [14]. On the other hand, gain-of-function mutations of Na_V_1.9 (encoded by *SCN11A*) have been linked to congenital pain insensitivity [34, 39, 43, 58] and to peripheral neuropathic pain [29, 30, 32, 33, 42, 47, 61].

Although the Na_V_1.8 variant p.V1287I present in our patient has been previously detected in genetic screenings aimed at identifying rare coding variants in genes associated with atrial fibrillation (AF) [2, 53], it was not considered causal for AF because it was detected in similar proportions in patients with a history of AF and in controls. Clinical symptoms other than AF were not studied, nor were the effects of alteration p.V1287I on Na_V_1.8 channel function.

Here we show that alteration p.V1287I confers both loss- and gain-of-function features to Na_V_1.8, characterized by depolarizing shifts of the voltage dependence of channel activation and inactivation. As a result, the overlap of activation and inactivation curves predicts a larger steady-state window current of the variant, specifically under depolarized conditions (Fig. 2D), a potentially pro-excitatory property which is expected to affect excitability of DRG neurons. Consistent with this, current-clamp recordings revealed that p.V1287I channels increase the amplitude and duration of individual action potentials and ultimately cause hyperexcitability of small-diameter DRG neurons, many of which are C-fiber neurons [12]. Thus, the data demonstrate that the mixed loss- and gain-of-function properties of Na_V_1.8-V1287I channels are translated into gain-of-function at the level of DRG neurons.

Qualitatively similar changes in channel gating, as described here for Na_V_1.8-V1287I, have been identified in Na_V_1.7 mutant channels that underlie PEPD in affected patients. The PEPD mutations characterized so far destabilize fast inactivation of Na_V_1.7, primarily by right-shifting the voltage dependence of steady-state channel inactivation, and most of them also right-shift the voltage dependence of channel activation [13, 20, 37, 56]. The clinical phenotype of PEPD is characterized by severe pain episodes affecting rectal, ocular, or submandibular body areas, combined with skin flashing but without involvement of hands or feet [13, 20, 31, 56], and is thus markedly different from the Na_V_1.8-V1287I-associated symptoms. However, with respect to symptoms and body areas affected, the Na_V_1.8-V1287I-associated disease shares some similarities with PE, a neuropathic disorder characterized by pain attacks, warmth and redness of body extremities, often triggered by heat or mild exercise [14, 57]. On the molecular level, PE is linked to a distinct group of Na_V_1.7 mutations that increase nociceptor excitability, predominantly by hyperpolarizing the voltage dependence of activation of Na_V_1.7 [14].

Based on a structural model of human Na_V_1.8 [36], mutation p.V1287I is localized to the intracellular part of transmembrane segment S5 in DIII of the channel, a region with high sequence conservation across Na_V_ subtypes (Fig. 1B). The intracellular DIII S4/S5 linker, located only a few residues upstream of the conserved valine (Fig. 1B), connects the voltage sensor of DIII (formed by transmembrane segments S1 to S4) to the channel pore and is likely part of the docking receptor for the inactivation particle of Na_V_ channels [49, 55]. Mutations in this region are therefore expected to affect channel activation or inactivation, or both processes. Our data indicate that p.V1287I increases the activation threshold and destabilizes inactivation of Na_V_1.8. In addition, some of the electrophysiologically confirmed Na_V_1.7-associated PEPD (p.V1298F, p.V1299D) and PE (p.P1308L, p.V1316A) mutations [5, 18, 37, 57] as well as one Na_V_1.9 mutation (p.V1184A) causing cold-aggravated peripheral pain [42] are located in or near the DIII S4/S5 linker, further emphasizing the disease-relevance of alteration p.V1287I in Na_V_1.8. Among these mutations, p.V1316A in Na_V_1.7 and p.V1184A in Na_V_1.9 are particularly interesting. Both affect the conserved valine corresponding to V1287 in Na_V_1.8, but unlike alteration p.V1287I in Na_V_1.8 they replace alanine for the conserved valine. The major gain-of-function property of Na_V_1.7-V1316A and Na_V_1.9-V1184A mutant channels is a hyperpolarized voltage dependence of channel activation [18, 42, 59], which contrasts with the right-shifted voltage dependence of activation and inactivation of Na_V_1.8-V1287I (this study).

Na_V_1.8-V1287A analyzed using an identical experimental setup revealed that the p.V1287A mutation shifted both the voltage dependence of channel activation and inactivation to more negative potentials, conferring gating alterations to Na_V_1.8 that are qualitatively opposite to those seen in Na_V_1.8-V1287I and more similar to those reported for Na_V_1.7-V1316A and Na_V_1.9-V1184A [18, 42, 59]. It is worth mentioning that the contrasting gating properties of mutants p.V1287A and p.V1287I correlate with the size (A: 88.6 Å^3^, V: 140.0 Å^3^, I: 166.7 Å^3^) and hydrophobicity (A: 1.8, V: 4.2, I: 4.5) [41, 60] of the side chain present at position 1287. While alanine (p.V1287A), which is smaller and less hydrophobic compared to valine, reduced the threshold for activation and inactivation of Na_V_1.8, incorporation of the larger more hydrophobic isoleucine increased activation and inactivation thresholds. However, the markedly hyperpolarized voltage dependence of activation of Na_V_1.8-V1287A (Δ*V*_*m*_: −12.1 mV) that is accompanied with a slow-down of channel deactivation (Fig. 1D–F) also suggests a gain-of-function mechanism for this variant. As revealed by current-clamp recordings of transfected murine DRG neurons, Na_V_1.8-V1287A channels caused hyperexcitability of the neurons characterized by a depolarized RMP of the neurons, a lowered voltage threshold for action potential takeoff (*V*_*th*_), and a marked increase in firing frequencies in response to depolarizing current injections. Qualitatively similar effects on DRG neuron excitability, namely, a depolarized RMP, a reduced threshold voltage at which action potential takeoff starts, and increased firing frequencies, were reported for the peripheral pain-associated mutation p.I1706V in Na_V_1.8, which also hyperpolarized the voltage dependence of channel activation but did not affect channel inactivation [35]. Therefore, the pro-excitatory properties of Na_V_1.8-V1287A channels are likely a direct consequence of their hyperpolarized activation.

Overall, our in vitro functional analyses showed that the p.V1287I variant significantly affects the function of sensory neuron-specific Na_V_1.8 channels. However, the frequency of p.V1287I in the general population is higher than would be expected for an ultra-rare monogenetic disorder with complete penetrance, which may indicate that the majority of carriers of the variant remain clinically inconspicuous because expressivity and thus severity of temperature sensation abnormalities might be variable among individuals. Yet unknown genetic or non-genetic factors are very likely involved in the manifestation of the disease. The relatively late but then sudden onset of the symptoms in the index patient may indicate the existence of triggering factors. However, in the present case, no triggering factor other than an infection coinciding with disease onset was identified. Incomplete penetrance and variable expressivity of associated symptoms are not uncommon and well documented for a number of Na_V_ variants. These include variants in Na_V_1.1 associated with epilepsy disorders [16, 21, 22, 46], Na_V_1.5 variants causing cardiac arrhythmia syndromes [23, 54], and variants in Na_V_1.7 underlying painful SFN [8, 10, 17].

In conclusion, the results of the present study suggest a relationship between the rare variant p.V1287I in Na_V_1.8 and altered peripheral temperature regulation. The Na_V_1.8 p.V1287I variant renders DRG neurons hyperexcitable by compromising channel inactivation as it was demonstrated previously for another Na_V_1.8 variant associated with painful small fiber neuropathy [28] and several Na_V_1.7 variants linked to PEPD [13, 20, 37, 56]. This study also shows that nonpainful peripheral paresthesias should be considered part of the clinical spectrum of Na_V_1.8-associated disorders.

## Data Availability

All data generated and analyzed during this study are included in this published article.
